# Epidemiology, morphometry, and phylogenetic analysis of *Haemonchus contortus* in small ruminants of Malakand division, Pakistan

**DOI:** 10.2478/helm-2025-0023

**Published:** 2025-11-26

**Authors:** M. I. Khan, W. Khan, A. Akbar, A. Ullah, A. Ali, A. U. Khan, P. R. De Los Ríos-Escalante

**Affiliations:** 1 Department of Zoology, University of Malakand, Khyber Pakhtunkhwa, Pakistan; 2 Department of Zoology, University of Swabi, Khyber Pakhtunkhwa, Pakistan; 3 Center for Biotechnology and Microbiology, University of Swat, Khyber Pakhtunkhwa, Pakistan; 4 Department of Zoology, Abdul Wali Khan University, Khyber Pakhtunkhwa, Pakistan; 5 Faculty of Natural Resources, Department of Biological and Chemical Sciences, Universidad Católica de Temuco, P.O. Box 15-D, Temuco, Chile

**Keywords:** Small ruminants, *Haemonchus*, Epidemiology, Morphometric, Cox-1, Phylogenetic analysis

## Abstract

*Haemonchus contortus* is a hematophagous nematode causing substantial economic losses to the livestock industry worldwide. This study aimed to investigate epidemiological determinants, morphometrics, and phylogenetic relationships of *Haemonchus* in small ruminants (goats and sheep) of Malakand division, Pakistan. Fecal samples (n=878) were randomly collected across four seasons (January to December, 2024) from six districts of the Malakand division to evaluate the prevalence and risk factors. Abomasa from 100 small ruminants were collected for morphometric and phylogenetic studies. Morphological identification was followed by DNA extraction from the adult worms and subsequent PCR amplification of the Cox-1 genetic marker. The overall prevalence of *Haemonchus* was 25.17 % (221/878), with significantly higher infection rates in small ruminants with poor health 36.19 % (38/105, P= 0.0003), those >5 years old 28.82 % (89/318, P= 0.0391), and untreated small ruminants 29.08 % (171/588, P= 0.0004), based on Chi-square test (P < 0.05), using bivariate analysis. Morphometric analysis revealed that the Haemonchus eggs were oval with an average length of 80.98 μm and a width of 45.04 μm. Male worms measured 11.9 – 13.8 mm long, while females ranged from 15 – 23.8 mm. Phylogenetic analysis based on the Cox-1 gene revealed a close genetic relationship between isolates from the Malakand division and those from Bangladesh, Pakistan, and Iran. Furthermore, the study identified two distinct haplotypes, providing further insights into the genetic variability within *Haemonchus* populations. This study highlights the significant burden of *Haemonchus* infections in the region, emphasizing health status, treatment, and age as key risk factors. The findings of this study provide a foundation for the development of effective control strategies against *Haemonchus* infections in small ruminants.

## Introduction

The livestock sector is a vital part of the national economy. In rural areas, 35 – 40 million people rely on livestock for 30 – 40 % of their income. Animal husbandry in Pakistan significantly contributes to livelihoods and poverty alleviation (Rehman *et al*., 2017; Riaz, 2022). Pakistan has 82.5 million goats and 31.9 million sheep, with Khyber Pakhtunkhwa hosting 9.50 million goats and 3.30 million sheep. Small ruminants are critical to the sustainability of agriculture in developing countries and contribute to a variety of socioeconomic activities across the world (Ministry of Finance, Government of Pakistan, 2022; Herrero *et al*., 2013).

Open grazing and extensive farming methods in sheep and goats increase the risk of worm infection and helminthiasis because they graze on worm larvae and eggs that have adhered to the grasses and pastures (Ijaz *et al*., 2009). *Haemonchus*, a barber pole or wireworm, infects millions of sheep and goats worldwide, causing massive economic losses (Carson *et al*., 2023; Akkari *et al*., 2013). Haemonchosis also negatively impacts the economy and drastically lowers the productivity of the affected sheep. In animals of all ages, adult worms cause edema, diarrhea, anemia, and occasionally even death by sucking the blood from the abomasum of sheep and goats (Osman *et al*., 2018; Santos *et al*., 2012; Ahmad *et al*., 2024). *Haemochus contortus* worms sucks around 0.05 ml of blood each day, causing anemia, meat and wool loss, and biochemical disruptions (Khattak *et al*., 2023; Rowe *et al*., 2008). *Haemonchus* produces up to ten thousand eggs daily, expelled in host feces, infecting millions of small ruminants worldwide (Qamar & Alkheraije, 2023).

*Haemonchus*, a new anthelmintic-resistant parasite, must be accurately identified to be controlled. Genetic exchange between small and large ruminants and shared pastures contributes to the transmission of resistant genes (Amarante *et al*., 2017). The traditional coprological diagnostic techniques for parasite species are challenging. Although morphology is helpful, it might not always resolve the taxonomic issues regarding species identification (Roeber *et al*., 2013; Wyrobisz *et al*., 2016). Because of their specificity, sensitivity, and precision, molecular techniques like polymerase chain reaction (PCR) help detect parasite species, particularly strongyle nematodes, and are, therefore, a valuable tool for taxonomic studies (Arbabi *et al*., 2020; Grillo *et al*., 2007).

A molecular marker, ITS-2, is used to identify closely related species. Similarly, the Cox 1 gene, mitochondrial DNA (mtDNA), is another molecular marker used for studying parasite genetic diversity due to its strong maternal lineage and rate of mutation (Mishra *et al*., 2021; Chrisanfova *et al*., 2021; Kandil *et al*., 2018).

According to previous studies conducted in Pakistan, the prevalence of *Haemonchus* ranges from 6.5 % to 87 % (Lashari & Tasawar, 2011; Sajid *et al*., 2000) as shown in Table 1. Small ruminants in Pakistan are affected by haemonchosis, which causes severe economic losses. Effective control requires a full understanding of the epidemiological factors driving disease progression, although there are no microscopic, morphometric, or phylogenetic studies available in the Malakand division. Therefore, this study aims to provide the first comprehensive study on *Haemonchus* in the Malakand division, which includes epidemiology, morphometric characterisation, and phylogenetic analysis. The work will use molecular and microscopic methods to resolve taxonomic ambiguities, accurately differentiate *Haemonchus* from closely related species and improve parasite identification procedures. It will also provide insights into the genetic patterns and evolutionary relationships of *Haemonchus* populations in Pakistan. The current study will increase our knowledge of Pakistan’s haemonchosis epidemiology, including its origin, transmission, and population structure, which could drive targeted control strategies and guide government actions to reduce the spread of the disease.

**Table 1.** Epidemiological spectrum of *Haemochus contortus* among sheep and goat populations in Pakistan.

Study area	Host	Sample type	Method	Samples	Positive	Prev. %	QAS	Reference
KPK	Goat & Sheep	Fecal	Mic & Mol	180	46	25.55	5	Ahmad *et al*., 2024
Sindh	Goat & Sheep	Fecal	Mic & Mol	800	88	11	4	Memon *et al*., 2024
KPK	Goat & Sheep	Fecal	Mic, Floa & Sed	1340	312	23.28	4	Irshad *et al*., 2023
KPK	Goat & Sheep	Fecal	Floa & MM	400	57	14.25	5	Khan *et al*., 2022
KPK	Goat & Sheep	Blood & Fecal	Mic	300	56	18.66	2	Ali *et al*., 2022
KPK	Goat & Sheep	Fecal	Mic	480	63	13.12	3	Aimen *et al*., 2022
KPK	Goat & Sheep	Fecal	Mic	315	71	22.53	3	Ruhoollah *et al*., 2021
Punjab	Goat & Sheep	Worm	Mic	200	105	52.2	3	Bibi *et al*., 2017
KPK	Sheep	Fecal	Floa, sed & MM	300	120	40	4	Jamil *et al*., 2016
Punjab	Goat & Sheep	Fecal	Mic & EPG	646	178	27.55	5	Qasim *et al*., 2016
KPK	Goats	Fecal	Mic	150	22	14.66	4	Nabi *et al*., 2014
Punjab	Goat & Sheep	Fecal	Floa & Sed	1000	133	13.3	4	Raza *et al*., 2014
Punjab	Goat	Fecal	Floa & Sed	100	20	20	3	Ayaz *et al*., 2013
Punjab	Goat & Sheep	Fecal	Floa, Sed & MM	480	137	28.54	4	Khalid *et al*., 2013
Balochistan	Sheep	Fecal	McMaster	1200	125	10.42	2	Razzaq *et al*., 2013
KPK	Sheep	Fecal	Mic & Floa	356	190	53.37	3	Ullah *et al*., 2013
Punjab	Sheep	Fecal	Mic, Floa & Sed	523	34	6.5	4	Lashari & Tasawar, 2011
Sindh	Goat	Fecal	Mic	1065	156	14.65	3	Akhter *et al*., 2011
Punjab	Sheep	Fecal	MM	333	259	77.7	3	Tasawar *et al*., 2010
Punjab	Goat & Sheep	Worm	K/Iden	4740	1604	33.83	1	Raza *et al*., 2009
Punjab	Goat & Sheep	Fecal	Mic, Floa & Sed	400	225	56.25	1	Gadahi *et al*., 2009
Punjab	Goat	Fecal	Mic, Floa & MM	300	84	28	2	Ijaz *et al*., 2008
Punjab	Sheep	Worm	Mic & K/Iden	960	591	61.5	3	Lateef *et al*., 2005
Punjab	Sheep	Fecal	Mic	10000	5477	54.77	2	Jabeen *et al*., 2000
Islamabad	Sheep	Fecal	Floa & MM	62	54	87	0	Sajid *et al*., 2000

1 Mic: Microscopy; Floa: Floatation; Sed: Sedimentation; MM: McMaster; K/Iden: Key identification; KPK: Khyber Pakhtunkhwa

## Materials and Methods

### *Study area* (Fig. 1)

**Fig. 1.**
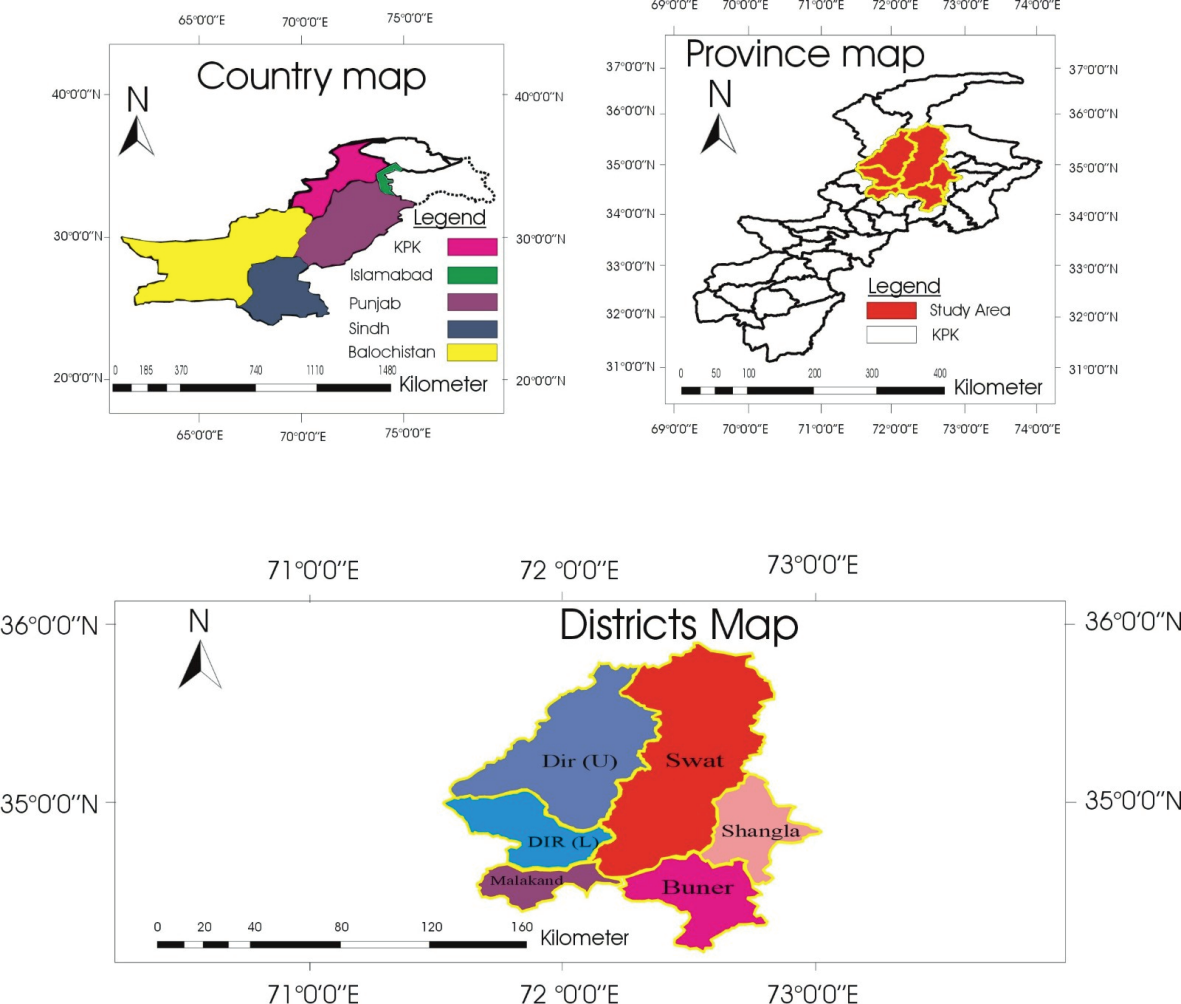
The study area map displays the geographical distribution of sampling locations in the Malakand Division of Khyber Pakhtunkhwa, Pakistan. It includes a national context map (country map), a provincial focus map (province map), and detailed district boundaries within the study area, highlighting Dir Upper, Dir Lower, Swat, Malakand, Shangla, and Buner. The map was designed and modified by the authors.

The study was conducted in Malakand division, Khyber Pakhtunkhwa, Pakistan (33° 59’ 59.89” N latitude and 72° 56’ 2.72” E longitude). Malakand Division is renowned for its extensive livestock farming and variety of agricultural activities. The region experiences average annual rainfall of 8.4 inches and temperatures ranging from -1°C in January to 42°C in July (Ali *et al*., 2021; Irshad *et al*., 2023).

### Collection of fecal samples and parasitological examination

In total, 878 (Goats: 398 and Sheep: 480) fecal samples were randomly gathered from sheep and goats across four seasons (January to December, 2024) from six districts of Malakand division, Pakistan, using a multistage random sampling design. Every fourth or eighth animal was sampled, depending on the flock size. To avoid repetition, no herd or animal was sampled more than once. Animals aged 3 months or older were included in the study; both healthy and unhealthy animals, regardless of treatment history, were sampled to allow comparative analysis. Pregnant, lactating and severely injured animals were excluded. Samples were collected directly from the rectum of sheep and goats. Comprehensive data encompassed animal category, time, gender, season, region, age, treatment history, dietary system, water source, and health status. Herd-level clustering was considered negligible, and no mixed effects modelling was applied due to the limited number of animals sampled per herd and a one-time visit design. Samples were examined at the Parasitology laboratory, Department of Zoology, University of Malakand, using the direct smear method under a microscope at 4X, 10X, and 40X. The *Haemonchus* eggs were identified based on the standard keys described by Mussa (2023) and Soulsby (1982).

### Adult worm collection

A total of 100 abomasa (50 goats, 50 sheep) were collected from local abattoirs of the study area and processed immediately in the laboratory. A total of 1200 adult *Haemonchus* were recovered using the procedures described by Kuchai *et al*. (2012). The worms were then cleaned in normal saline and washed in phosphate-buffered saline (PBS; pH=7.4) containing ampicillin (100 mg/ml) and cloxacillin (50 mg/ml) to inhibit microbial growth. Subsequently, *Haemonchus* was identified based on morphological characteristics using the key described by Soulsby (1982). Parasites were stored at -20°C in 70 % glycerin alcohol solution for molecular analysis.

### Morphometric characterization of eggs and adult worms

For morphometric characterization, 35 samples of *Haemonchus* eggs and 126 (76 females and 50 males) samples of adult worms were randomly selected for measurement. Parasitic eggs and adult worm measurements were conducted using a compound microscope (Olympus BX 53, Tokyo, Japan). Body length, body width, esophageal length, spicule length, gubernaculum length, vulva to posterior end distance, and anus to posterior end distance were measured in millimeters. In addition, the egg morphology, including the egg’s length, width, and shape, was evaluated as described by Lichtenfels *et al*. (1994) and Soulsby (1982).

### Phylogenetic analysis

### DNA Extraction

Genomic DNA was extracted from individual *Haemonchus* samples using a commercial DNA extraction kit (Qiagen, Hilden, Germany), following the manufacturer’s guidelines.

### PCR amplification

To amplify the mitochondrial COX-1 gene for phylogenetic analysis, a polymerase chain reaction (PCR) was performed following Kandil *et al*. (2018). The primer sets used were LCO1490 (5' GGTCAACAAATCATAAAGATATTGG 3') as a forward primer and HCO2198 (5' TAAACTTCAGGGTGACCAAAAAATCA 3') as the reverse primer. Thermocycling conditions: 95°C for 5 minutes, 30 cycles of 95°C for 60 seconds, 55°C for 45 seconds, 72°C for 45 seconds, and a final extension at 72°C for 7 minutes. The amplified Cox-1 gene PCR products were visualized through agarose gel electrophoresis, as described by Ndosi *et al*. (2023).

### Sequencing and Phylogenetic tree construction

The amplicons were confirmed through gel electrophoresis. Two amplified DNA products (one each from goats and sheep samples) were randomly selected, purified, and sequenced at Macrogen Inc. in Seoul, South Korea. The sequences were assembled and aligned using ClustalW multiple alignment implemented in MEGA11. Sequence analysis was performed using BLASTn algorithms and databases from the National Center for Biotechnology Information (NCBI) database. A phylogenetic tree was constructed using a neighbor-joining method implemented in MEGA 11.

### Statistical analysis

Data were analyzed using SPSS 20 and GraphPad Prism V.7.0. Correlation between risk factors and *Haemonchus* prevalence was assessed through bivariate analysis using Chi-square tests. Descriptive statistics (standard deviation, error, and variance) were calculated. A p-value < 0.05 indicated statistical significance.

## Ethical Approval and Conflict of Interest

The University of Malakand’s advanced studies board approved the study protocols (UOM/Admin/2024/501). Informed consent was obtained from the owners of small ruminants that participated in the study. The authors declare that there are no conflicts of interest related to this study.

## Results

### Prevalence and Risk Factors

During the study period from January to December (2024), 878 fecal samples (Goats: 398 and Sheep: 480) were collected randomly from goats and sheep across six districts of the Malakand Division, namely (Swat, Shangla, Dir L, Dir U, Buner, and Malakand). Two hundred twenty-one (221) small ruminants tested positive for *Haemonchus*, resulting in an observed total prevalence of *Haemonchus of* 25.17 %. Sheep had a slightly greater prevalence of haemonchosis (26.04 %) than goats (24.12 %), although the difference was not statistically significant (p > 0.05) (Table 2).

**Table 2.** Overall prevalence of *H. contortus* in goats and sheep in Malakand division.

Host	Sample	Positive	Percentage	P-value
Goats	398	96	24.12	0.51379^ns^
Sheep	480	125	26.04	
Total	878	221	25.17	

ns P-value is non-significant

In the current study, female sheep exhibited a higher prevalence of *Haemonchus* (27.86 %) compared to male sheep (23.5 %), as well as female goats (25.22 %) and male goats (22.62 %). However, this difference was not statistically significant (p>0.05).

Goats and sheep were categorized into three age groups: less than 1 year, 1 to 5 years, and greater than 5 years. *Haemonchus* infection rates varied significantly by age group (p < 0.05). Specifically: Younger animals (<1 year) had the highest infection rates, with 29.25 % in goats and 27.13 % in sheep, followed by older animals (>5 years) years Sheep 28.82 % and goats 27.03 % while animals aged (1 – 5) years had the lowest infection rates: 17.36 % in goats and 22.65 % in sheep.

This study was conducted across six districts of the Malakand division: Swat, Shangla, Dir (L), Dir (U), Malakand, and Buner. The prevalence of haemonchosis varied across districts, with Swat (26.22 %), Shangla (24.44 %), Dir (L) (26.28 %), Dir (U) (23.08 %), Malakand (26.25 %), and Buner (24.06 %). However, these differences were not statistically significant (p>0.05). Furthermore, the prevalence of haemonchosis was highest in grazing sheep (27.84 %) and goats (26.19 %) compared to stall-fed sheep (24 %) and goats (21.81 %), though the difference was not statistically significant (p>0.05).

Concerning health status, a statistically significant variation (p<0.05) was observed; the highest prevalence of haemonchosis was observed in poor goats (36.73 %) and sheep (35.71 %), followed by moderate sheep (30.11 %) and goats (26.97 %). The lowest prevalence rate was found in healthy goats (18.78 %) and sheep (20.59 %).

In addition, a high degree of Haemonchosis was observed in open water drinking goats (28.46 %) and sheep (28.39 %), followed by stream water sources drinking sheep (28.93 %) and goats (25.56 %) compared to other water sources like tap and bore water, the difference was not statistically significant (p>0.05). The prevalence of Haemonchosis in goats and sheep was further evaluated in different seasons. The highest prevalence was found in summer (27.69 %), while the lowest prevalence was recorded in winter (22.11 %), though this difference was not statistically significant (p>0.05).

Furthermore, statistically significant (p<0.05) variations between treated and untreated small ruminants were observed. The highest infestation was observed in untreated sheep (29.79 %) and goats (28.19 %), while the lowest prevalence was found in treated goats (16.55 %) and sheep (17.88 %), Table 3.

**Table 3.** Prevalence of *Haemonchus contortus* in small ruminants concerning various risk factors in Malakand division.

Risk Factors	Variables	Total samples	Total positive cases	Total prevalence (%)	Goats Positive (%)	Sheep Positive (%)	P-value
Gender	Male	368	85	23.1	22.62	23.50	0.2292^ns^
	Female	510	136	26.7	25.22	27.86	
Age	<1 years	235	66	28.1	29.25	27.13	0.0391*
	1-5 years	325	66	20.3	17.36	22.65	
	>5 years	318	89	28	27.03	28.82	
Area	Swat	164	43	26.2	23.19	28.42	0.9815^ns^
	Shangla	135	33	24.4	23.61	25.40	
	Dir (L)	156	41	26.3	25.00	27.50	
	Dir (U)	130	30	23.1	23.21	22.97	
	Malakand	160	42	26.2	25.76	26.60	
	Buner	133	32	24.1	23.73	24.32	
Grazing system	Grazing	465	126	27.1	26.19	27.84	0.1629^ns^
	Stall-fed	413	95	23	21.81	24.00	
Health condition	Healthy	435	86	19.8	18.78	20.59	0.0003*
	Moderate	338	97	28.7	26.97	30.11	
	Poor	105	38	36.2	36.73	35.71	
Drinking water source	Open	285	81	24.4	28.46	28.39	0.1729^ns^
	Bore	187	40	21.4	20.45	22.22	
	Stream	211	58	27.5	25.56	28.93	
	Tap water	195	42	21.5	20.00	22.86	
Season	Autumn	205	48	23.4	21.98	24.56	0.5016^ns^
	Winter	190	42	22.1	20.51	23.21	
	Spring	223	59	26.5	25.23	27.59	
	Summer	260	72	27.7	27.05	28.26	
Treatment	Treated	290	50	17.2	16.55	17.88	0.0004*
	Untreated	588	171	29.1	28.19	29.79	

ns P-value is non-significant

* statistically significant

### Morphometric characterization of Haemonchus eggs

**Fig. 2.**
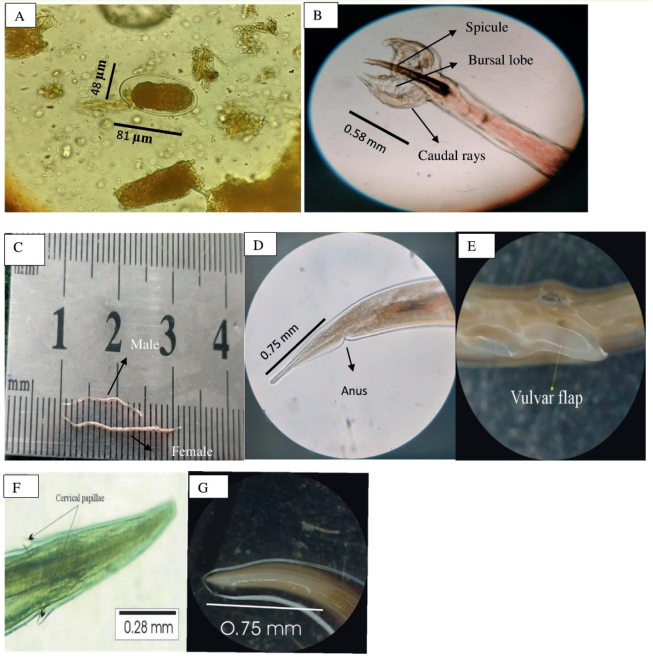
Morphological features of *Haemonchus contortus*. (A). Egg containing morula, showing measured length and width (μm); (B). Male bursa highlighting spicules and caudal rays, with spicule length (mm); (C). Adult male and Female *H. contortus* aligned on a calibrated scale to show total body length (mm); (D). Posterior end of female showing anus and measured tail length (mm); (E). Vulvar flap of female *Haemonchus*; (F). Cervical papillae with recorded length (mm); (G). Oesophagus of *H. contortus* showing total length (mm)

The present study randomly selected 35 positive samples from different small ruminants for egg morphometric characterization. *Haemonchus* eggs were oval, with equal poles and divided cells that did not fill the egg cavity (Fig. 2A). The average length of the eggs was 80.98μm, ranging from 63μm to 99μm. Egg width ranged from 36μ to 61μ, with an average width of 45.04μ. (Table 4).

**Table 4.** Morphometric characteristics of *H. contortus* egg sizes (in μm).

Shape	Statistical index	Length	Width
		μm	μm
Oval	Average	80.98	45.05
	Maximum	99	61
	Minimum	63	36
	Standard error	1.6016	1.43
	Standard deviation	9.4749	8.49
	Sample variance	89.7754	72.14
	N	35	35

1 N: Number of eggs examined

### Morphology and morphometry of adult worms

A total of 1200 *Haemonchus* worms were recovered from 100 abomasums collected from the different abattoirs of the study area. One hundred twenty-six worms (76 females and 50 males) were randomly selected (from goats and sheep) for morphometric analysis. The results are summarized in Table 5. The anterior end was broad and blunt, with a small buccal cavity featuring a visible tooth protruding from the dorsal wall. The cervical papillae of both male and female worms possessed a spine-like protrusion on its outside that ran almost parallel to the longitudinal cuticular ridges. The body also had transverse striations, which were visible.

**Table 5.** Morphometric measurement (in mm) of *H. contortus* isolated from goats and sheep.

Particulars	Male worms (n= 50)	Female worms (n= 76)
Body length (mm)	11.9 – 13.8	15 – 23.8
Body width (mm)	0.23 – 0.38	0.29 – 0.38
Cervical papillae (mm)	0.28 – 0.38	0.24 – 0.42
Spicule length (mm)	0.44 – 0.78	–
Length of esophagus (mm)	0.61 – 0.92	0.72 – 1.22
Gubernaculum length (mm)	0.09 – 0.18	–
Distance from vulva to posterior end (mm)	–	0.68 – 0.82
Distance from Anus to posterior end (mm)	–	0.29 – 0.35

### Male Morphometry

Male worms were smaller than females, measuring 11.9 to 13.8 mm in length and 0.23 to 0.38 mm in width (Fig. 2C). The cervical papillae ranged from 0.28 to 0.38 mm, whereas the esophagus measured 0.61 to 0.92 mm. A bursa with two large lateral lobes and a small dorsal lobe was observed at the tail (Fig. 2B). The asymmetrically branched dorsal ray divided near the tip, creating a short external branch. Two 0.44 to 0.78 mm long spicules with a bulb and pore toward the end were supported by a spindle-shaped gubernaculum tapered toward the tail end and measured 0.09 to 0.18 mm.

### Female Morphometry

The length of female *Haemonchus* measured 15 – 23.8 mm while the width was 0.29 – 0.38 mm (Fig. 2C). The cervical papillae measured 0.24 – 0.42 mm (Fig. 2F), while the esophagus was lengthy and club-shaped, measuring 0.72 – 1.22 mm (Fig. 2G). Female *Haemonchus* has a slender, spineless, straight, and pointed tail. The characteristic ‘barber pole’ appearance, resulting from the coiled uterus and intestines, was observed. The vulva is located in the posterior portion of the body, approximately 0.68 – 0.82 mm from the tail end, and is covered by a large vulvar flap (Fig. 2E). The anus is located 0.29 – 0.35 mm from the posterior end (Fig. 4D).

### Phylogenetic Tree

**Fig. 3.**
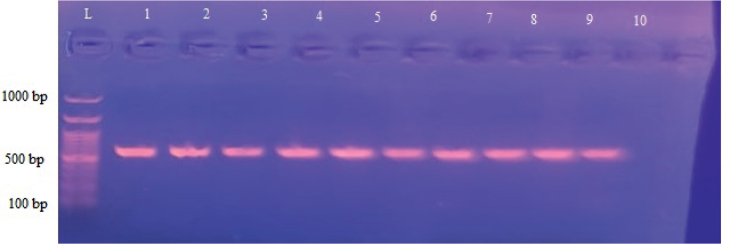
Agarose gel electrophoresis showing PCR amplification of COX region of *H. contortus*.

Following DNA extraction and COX-1 gene amplification, the PCR products of two *Haemonchus* isolates from Malakand Division were examined under UV light, producing an amplicon of approximately 666 bp (Fig. 3). The Neighbor-Joining method was utilized to construct a phylogenetic tree comparing the two isolates from the current study with 19 published sequences from various regions, including Pakistan, Bangladesh, Iran, Brazil, USA, New Zealand, Australia, and the UK, as well as out-group nematode species.

**Fig. 4.**
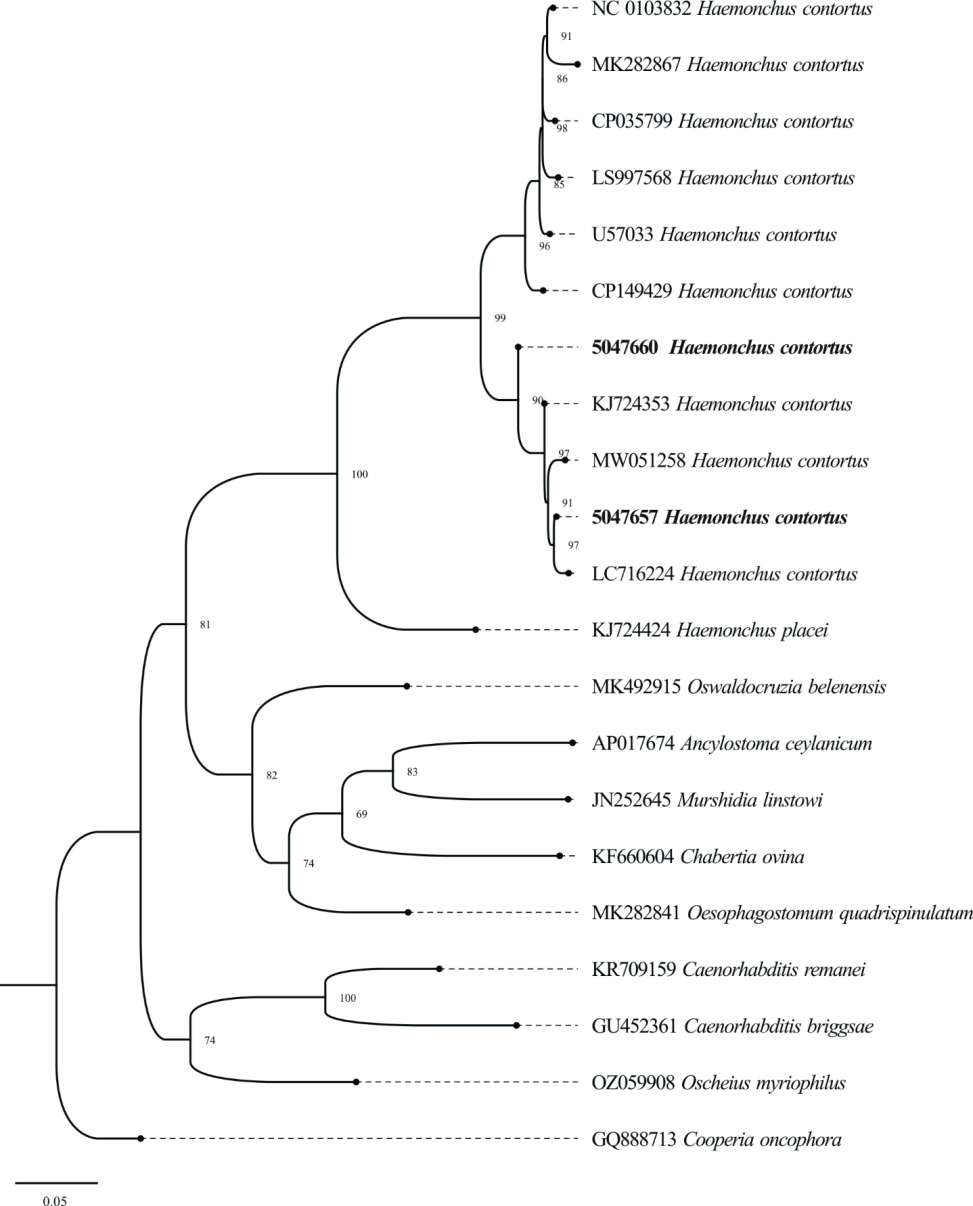
Phylogenetic tree of *H. contortus* in Goats and Sheep using Cox-1 marker. The constructed tree illustrates the genetic links between the isolates of the current study and reference sequences from other areas. Bootstrap values are given at branch points representing the group’s confidence levels.

Phylogenetic analysis revealed two distinct haplotypes in the present study isolates. One isolate (5047657) was grouped with isolates from Pakistan (724353), Bangladesh (LC716224), and Iran (MW051258), suggesting genetic similarity with the isolates from neighboring regions. The other isolate (5047660) formed a separate haplotype (Fig. 4). These findings indicate the presence of two distinct genetic variants of *Haemonchus*, which may reflect intraspecies variation or localized adaptation within *Haemonchus* populations in the Malakand division.

## Discussion

*Haemonchus*, a parasite that affects both the health and production of animals, poses a serious challenge to the livestock industry and results in substantial economic losses (Arsenopoulos *et al*., 2021). Given the significance of haemonchosis, this study aimed to evaluate the prevalence, risk factors, morphometric characteristics, and phylogenetic analysis of *Haemonchus* in small ruminants of the Malakand Division. Previous studies were reviewed and summarized to provide a contextual background on the epidemiology of haemonchosis in Pakistan (Table 1). The present study revealed that the prevalence of Haemonchus in small ruminants of Malakand division was 25.17 %, which falls within the broad range reported in earlier studies (6.5 % to 87 %; Table 1). Various factors, such as management strategies, environmental variability, sample size differences, and seasonality in the research region, could be responsible for the observed fluctuation in prevalence. The parasite density may be affected by grazing patterns, socioeconomic factors, and overuse of anthelmintics (Lashari & Tasawar 2011).

The current study found that the overall sex-wise prevalence of haemonchosis in small ruminants was higher in females (26.66 %) compared to males (23.09 %), p>0.05. According to similar studies conducted by Ahmad *et al*. (2024), Raza *et al*. (2014), and Brik *et al*. (2019), females are more prone to parasitism due to reproductive stress and compromised immune systems, which may account for the greater occurrence of haemonchosis. However, the current study contradicts the findings of Rinaldi *et al*. (2015) and Mohamed *et al*. (2024), who reported that males had a higher prevalence of *Haemonchus* than females, suggesting that local factors may also influence the susceptibility.

Age-specific prevalence showed higher infection rates in the oldest (>5 years) (27.98 %) and youngest (<1 year) (25.53 %) age groups. These results align with the findings of Ullah *et al*. (2013) and Adduci *et al*. (2022), who also reported higher rates of *Haemonchus* infection in younger and older age groups. The higher infection rates in older and younger age groups may be attributed to weaker immunity. Targeted control measures for these vulnerable age groups could help reduce the parasite burden.

Risk factor analysis highlighted the grazing system as a significant determinant of infection, with a higher prevalence (27 %) in grazing small ruminants than in stall-fed small ruminants (23 %). Our results align with those of Shahzad (2016). Greater exposure to infectious larvae on contaminated pastures may be the cause of the higher prevalence of haemonchosis in grazing animals.

Similarly, access to contaminated water sources and poor body condition were associated with higher infection rates. The highest infection rate was found in small ruminants with poor body condition (36.19 %) and those drinking from streams and open water sources (28.42 % and 27.48 %, respectively). These results highlight the role of nutrition, water hygiene, and grazing management in controlling haemonchosis. The present study is consistent with earlier findings of Shahzad (2016), Sangma *et al*. (2012), and Irshad *et al*. (2023).

In sheep and goats, seasonal prevalence rates of *Haemonchus* showed that the highest infection rates were observed in the spring (27.59 %) and summer (28.26 %). Monsoon rains, dense vegetation, and frequent temperature changes may be the cause of the increased prevalence of Haemonchosis in small ruminants during these seasons. These findings align with those of Irshad *et al*. (2023).

According to the study, the prevalence of *Haemonchus* was significantly higher in untreated small ruminants (29 %) than in treated ones (17 %). These results are in line with those of Raza *et al*. (2007), Irshad *et al*. (2023), and Ijaz *et al*. (2009), who similarly reported lower infestation rates in treated animals. The high prevalence of Haemonchosis in the research area’s animal population could result from negligence and mistreatment.

The eggs of *H. contortus* in the current study were oval, with an average length of 80.98 μm and a width of 45.0 μm. The findings of the current study are consistent with Soulsby (1965), who found *H. contortus* eggs to measure around 74×44 μm. The findings of the current study contrast with those of Thienpont (2003) and Inder *et al*. (2010), who reported larger sizes of *H. contortus* eggs, measuring 83.7×45.2 μm and 94.7×58.3 μm, respectively. Variations in egg size among these studies may have been due to host species, geographical location, environmental conditions, or the methodology employed for measurement.

The findings of the current study provide an essential understanding of *Haemonchus*’ morphometric features, which are necessary for parasite identification and classification. Morphometric measurements of *H. contortus* in the current study showed both consistency and variations with prior studies. The body length of male worms (11.9 – 13.8 mm) was slightly greater than reported by Nath *et al*. (2021) and Kuchai *et al*. (2012), although shorter than that documented by Bibi *et al*. (2017). Male spicule lengths (0.44 – 0.78 mm) were consistent with the findings of Soulsby (1982) and Bibi *et al*. (2017), although gubernaculum and esophagus lengths were typically shorter than previously documented. The body lengths of females (15 – 23.8 mm) were comparable to the findings of Kuchai *et al*. (2012) but were shorter than those reported by Bibi *et al*. (2017). The lengths of the esophagus and the distances from the vulva and anus to the posterior end were also lower than those reported by Nath *et al*. (2021).

The consistency in morphometric parameters between the current study and prior studies verifies that *Haemonchus* is accurately identified in small ruminants of the Malakand Division. The minor variations between the present study and previous findings may be due to host-related and geographic factors that influence the morphology of parasites. However, differences have little impact on the overall identification of *Haemonchus*.

Phylogenetic analysis of *Haemonchus* isolates of the Malakand Division, using COX-1 gene sequences, revealed genetic variation. Genetic divergence between isolates 5047657 and 5047660, as revealed by phylogenetic analysis, may suggest intraspecies diversity that is influenced by host differences, geographic location, or environmental stress in the Malakand. The same findings have been reported by Yin *et al*. (2013), Parvin *et al*. (2024), and Moudgil *et al*. (2022), who observed that *Haemonchus* often forms two to three clades based on host-associated and geographic variations.

### Limitation

The primary limitation of this study was time constraints, which resulted in restricted sampling across districts and limited sequencing for genetic diversity analysis. Future studies should focus on expanding sampling and sequencing efforts to understand better the genetic diversity of *Haemonchus* in the area.

## Conclusion

The current study concluded that *Haemonchus* is prevalent in Pakistan, particularly in the Malakand Division and is significantly associated with health status, prior anthelmintic treatment and host age. Phylogenetic analysis revealed two genetically distinct isolates forming separate clades, indicating genetic diversity within the isolates. This study contributes to a better understanding of epidemiological and genetic patterns of *Haemonchus*, which may aid future research on parasite management. Future studies should explore additional genetic markers and geographic regions in order to enhance the understanding of *Haemonchus* diversity and epidemiology.
